# Relationships between digital engagement and the mental health of older adults: Evidence from China

**DOI:** 10.1371/journal.pone.0308071

**Published:** 2024-08-06

**Authors:** Ziqiong Liu, Ziwei Li

**Affiliations:** 1 School of Economics and Management, Anhui University of Chinese Medicine, Hefei, Anhui, China; 2 School of Public Administration, Guangdong University of Finance, Guangzhou, Guangdong, China; BRAC Business School, BRAC University, BANGLADESH

## Abstract

Based on the theory of socio-emotional selectivity, this study examines the effect of digital engagement on the mental health of older individuals using data from the 2018 China Longitudinal Aging Social Survey (CLASS). The results show that digital engagement has a significant effect on the mental health of older individuals, manifested by a decline in depression and an enhancement of cognitive abilities. The results are robust by Using instrumental variables to solve endogenous problem and the propensity score matching method to solve selective bias. The analysis of heterogeneity demonstrates that digital engagement can significantly reduce the depression level of older people without chronic diseases and at lower ages and promote the cognitive ability of older adults without chronic diseases and older adults of higher ages. Mechanistic analysis shows that digital engagement can reduce depression in older adults by alleviating loneliness and improving emotional well-being and cognitive performance by enhancing social support. Therefore, digital engagement gives older people a more positive emotional experience and more social support and thereby improves mental health, delivering proactive answers to the problems an aging population brings.

## 1. Introduction

China is one of the fastest aging countries in the world and has the highest population of senior citizens. According to the seventh national census, which was carried out in 2020, 13.5% of the population is over the age of 65, while 18.7% of the population is over the age of 60 [1]. Alarmingly, China’s older population faces considerable challenges concerning their mental health. For instance, approximately one-third of Beijing’s older population has symptoms of depression [2]. Hence, it is crucial to prioritize the enhancement of mental health support for the seniors and share China’s experiences in effectively addressing population aging.

Digitization arrival is inevitable with aging population growth [3]. Digital innovations are essential for advancing society and changing people’s lifestyles, making it necessary for people to constantly improve their digital literacy to successfully adjust to the developing digital era [4, 5]. Unfortunately, due to their lack of digital capabilities, older generation get stuck in the digital gap and must deal with prejudice, exclusion, and other digital impediments [6]. As a result, they end up being digital refugees or ostracized in the digital world. The recent COVID-19 pandemic outbreak has highlighted the need for older adults to be more engaged online [7]. The widespread use of mobile internet, smartphones, and other electronic devices and applications in every field of society has raised the significance of digital technology as a necessary tool for daily tasks, emphasizing the pressing need to equip older people with digital skills [8, 9]. By the end of 2022, China’s population of Internet users had reached 1 billion, with a penetration rate of 75.6%, according to the Statistical Report on Internet Development in China. However, only 14.3% of the older population was active online. An urgent public issue that requires comprehensive attention and multilevel solutions at the individual, familial, communal, and societal levels is the difficulty of bridging the "digital divide" for the seniors and facilitating their involvement in the digital world [10, 11]. With China becoming an aging society, it is critical to minimize the digital barriers that older adults must conquer, explain the relationship between their use of technology and their mental health, encourage the seniors to develop their use of technology in a way that is harmonious with their mental health and resolve the conflict between societal digitization and population aging. Collectively, these goals constitute a very substantial research project.

According to socio-emotional selectivity theory, older persons’ emotions and affective reactions are complex and change depending on the circumstances [12]. As individuals enter old age, their perceptions of time tend to become more limited as they grow older, and older people are more likely to believe that time will be limited in the future. As a result, older people prefer emotional targets that may be achieved quickly. This means bringing their emotional state into focus, delving into the significance of their emotions, and entirely embracing emotional fulfillment [13]. For older individuals, digital engagement means more than just owning digital devices; it also means using those devices and having basic digital ability [14]. Accordingly, concerning a theoretical perspective, older adults’ involvement in the digital world not only makes it easier for them to learn, gather information, and be exposed to new experiences but also provides them with tools and platforms to focus on their emotional well-being [15].

Existing studies have predominantly focused on investigating the effects of internet use on the health of older adults. However, limited attention has been given to the concept of digital engagement, and a consensus has yet to be reached on this matter. Some scholars argue that internet utilization among older adults positively impacts their mental well-being. For instance, Moult et al. (2019) suggest that internet usage contributes to the effective self-management of health-related issues, enabling older adults to access relevant information [16]. Moreover, internet use can mitigate feelings of loneliness, enhance overall well-being, and improve mental health among older individuals [17]. Supporting this viewpoint, Cotten (2014) conducted research that revealed a lower prevalence of depression among older adults who engage in internet activities [18]. Similarly, Boekel’s (2017) study on internet usage by older individuals in the Netherlands indicated that such use facilitates the development of social capital [19]. It enables older adults to expand or sustain social connections, gain access to information, and reduce feelings of isolation. Cotten (2012) discovered that Internet usage can assist older individuals in surmounting temporal and spatial constraints [18]. The heightened frequency of social interactions facilitated by the Internet has the potential to alleviate loneliness among older adults, leading to a positive relationship on their emotional well-being and enabling them to attain an improved psychological state. Nevertheless, certain scholars maintain contrasting perspectives, contending that Internet utilization may not be advantageous for the mental health of older individuals. They argue that technological obstacles could induce anxiety in older individuals, and they may also be susceptible to misinformation, thereby resulting in potential harm to their mental well-being [20–22].

This study uses 2018 CLASS data to examine the relationship between digital engagement and the mental health of the senior population, which is based on the theory of socio-emotional selectivity. The primary objective is to address the following questions: (1)Does the digital engagement have a relationship with the mental health of older adults? If so, is the result reliable and consistent? (2)Is there variability in the relationship between digital engagement and the mental health of older adults across different groups?(3) What are the specific channels and mechanisms through which digital engagement establishes a relationship with the mental health of the elderly? The answers to these questions will not only enhance our comprehension of the relationship between digital engagement among older adults and mental health outcomes but also hold significant implications for the development of public policies aimed at improving the mental well-being of the older population.

## 2. Models and methods

### 2.1 Data source

Data from the 2018 China Longitudinal Aging Social Survey (CLASS) were used in this study. A nationwide longitudinal sociological survey project is managed and carried out by China’s Renmin University. CLASS’s main goal is to gain a thorough grasp of the aged population’s position. Twenty-eight provinces—including municipalities and autonomous regions—are covered by the survey. All procedures performed in the study involving human participants were in accordance with the ethical standards of the institutional and/or national research committee and with the 1964 Declaration of Helsinki and its later amendments or comparable ethical standards. The survey was also conducted within Articles 38, 39, and 40 of the Constitution of the People’s Republic of China and the legal framework governed by Chapter I, Article 9 from the Statistics Law of the People’s Republic of China. Therefore, the study was not reviewed by an ethics committee. Verbal informed consent was obtained from all individual participants included in the study. The design of this survey adhered to Articles 38, 39, and 40 of the Constitution of the People’s Republic of China and the legal framework governed by Chapter I, Article 9 from the Statistics Law of the People’s Republic of China. Verbal informed consent was considered acceptable, and this was not reviewed by an ethics committee. Moreover, the interviewer also documented more detailed information on the process of obtaining informed consent, which included whether participants agreed to participate in this study, the time of agreement, the reasons for not agreeing, etc. Details of informed consent were stored by the Institute of Gerontology and the National Survey Research Center at Renmin University of China. The survey includes many elements linked to the individual, familial, and social characteristics of the seniors using a hierarchical multistage randomly selected approach. This includes analyzing the digital engagement of the elderly and their mental health status. Therefore, the survey provides a wide national sample, allowing for a thorough analysis of senior citizens’ digital involvement and mental health. After preliminary combing of original data, the samples with missing values were deleted, and only analytical samples with complete information were retained, a total sample size of 9,278 participants was obtained to fulfill the research needs of this paper, and all variables were well controlled.

### 2.2 Variables

This chapter focuses on the mental health of older individuals, with the factors of concern including symptoms of depression and cognitive abilities. The CES-D simplified scale in CLASS was used to measure the symptoms of depression in this study. The questionnaire was designed to include the following relevant questions during the past week:

1. Have you experienced a positive mood?
2. Have you felt sadness?
3. Have you perceived your days as good?
4. Have you experienced a lack of appetite?
5. Have you had difficulty sleeping?
6. Have you felt a sense of worthlessness?
7. Have you felt a lack of purpose or things to do?
8. Have you found life to be enjoyable or interesting?
9. Have you felt a lack of companionship?
10. Have you felt ignored by others?
11. Have you felt socially isolated?

The answers to these questions were coded as "No," "Sometimes," or "Often," with corresponding scores of 0, 1, and 2. It is worth noting that positive questions (1, 3, 8) were scored backward. A higher total depression score means a greater tendency or degree of depression in older individuals. Older individuals’ cognitive ability is composed of three sections: basic cognitive ability (connected to common sense), memory, and calculation. A set of five life-related common sense questions is used to measure basic cognitive abilities, with one point provided for each correct answer. Memory is evaluated by repeating three words twice, with one point scored for each correctly recalled word, for a total of six points.

The main explanatory variable examined in this paper is older adults’ digital engagement. The discussion on the digital engagement of older individuals cannot be separated from the concept of the digital divide among this age group. The presence of a digital divide among older adults acts as an obstacle to their digital engagement, and the two factors are inherently opposing. While the term "digital divide" holds significant social and academic importance, it is not without flaws, as it may promote techno-deterministic thinking [23]. On the other hand, the concept of digital engagement among older adults provides a more comprehensive understanding of the issue, as it reflects the varying levels of digital technology adoption among individuals and offers potential solutions [24].

Bridging the objective digital divide is a fundamental prerequisite for ensuring the digital engagement of older adults [6, 25]. Consequently, digital engagement among older people refers to their ability, within a digital society, to overcome the three-tiered digital divide, possess a certain level of digital literacy, and effectively acquire and utilize digital devices. In this paper, digital engagement variables are constructed from three dimensions: access to digital devices for older individuals, utilization of digital devices, and basic digital literacy. Access to digital devices encompasses two aspects: "availability of network coverage" and "possession of a smartphone." Utilization of digital devices is measured by the older adults’ internet usage, while basic digital literacy is determined by their attainment of a basic education level. The specific indicators within each dimension are equally weighted to calculate the final composite index. The weights for access to digital devices, digital use and digital literacy are each set at 1/3. The calculation formula is 1/3*(1/2*(internet coverage)+1/2*(possession of a smartphone))+1/3*(digital use)+1/3*(digital literacy), and all of the indicators are binary systems. A higher value on this index indicates greater digital engagement among older adults.

Control variables included influencing factors related to individual characteristics, individual experiences, family characteristics, and community characteristics of older adults. Individual characteristics encompassed age, gender, marital status, and household registration status of older individuals. Individual experiences included indicators such as experiencing a major accident in the past year, childhood hunger, and parental status at age ten. Family characteristics comprised child support and family dynamics, while community characteristics encompassed the prevailing social security conditions. For precise definitions and measurement methods of these variables, please refer to Table 1.

**Table 1:** Variable descriptive statistics.

Variable	Variable definition	Average	Standard Deviation
Explained variable			
Depression	Calculated according to the scale, the higher the score, the greater the degree of depression	8.629	3.175
Cognitive ability	Basic cognition + computing power + memory comprehensive assessment	12.409	3.059
Explanatory variable			
Digital engagement	Weighted gains from digital access, digital use and digital literacy for older adults	0.405	0.322
Digital devices	Yes = 1, no = 0	0.321	0.400
Digital use	Yes = 1, no = 0	0.191	0.393
Digital literacy	Yes = 1, no = 0	0.703	0.457
Individual characteristics			
Age	Chronological age of the older adults	71.234	7.289
Gender	Males = 1, females = 0	0.501	0.500
Marriage	Married = 1, unmarried = 0	0.696	0.460
Registration	Rural = 1, urban = 0	0.521	0.500
Individual experiences			
Major accidents	Yes = 1, no = 0	0.175	0.380
Childhood hunger	Experience hunger in childhood = 1, no hunger experience in childhood = 0	0.662	0.473
Parents survive	Yes = 1, no = 0	0.894	0.308
Family status			
Child support	Yes = 1, no = 0	0.873	0.333
Home light	Home dim light = 1, good light = 0	0.117	0.321
Community characteristics			
Community policing	The degree of satisfaction of the elderly with community policing	3.818	0.807
Instrumental variable			
IPv4 ratio	Provincial IPv4 ratio	0.040	0.055
Possible channel variables			
Loneliness	Yes = 1, no = 0	0.466	0.499
Social Support	Number of support from relatives and friends	4.449	2.041

### 2.3 Methods

The mental health index for older individuals, developed in this study, is represented as a continuous variable. Therefore, in the benchmark regression analysis, the initial estimation is performed using the ordinary least squares (OLS) method. The fundamental formula for OLS is as follows:

$${MHealth}_{i}=\alpha_{1}+\alpha_{2}{Digital}_{i}+\beta X_{i}+\varepsilon_{i}$$ (1)

*Mhealth*_*i*_ in Eq (1) indicates mental health in older individuals. *Digital*_*i*_ is the digital engagement of the older people surveyed and is the explanatory variable that this article focuses on. *X*_*i*_ represents all other control variables, *ε*_*i*_ is the random error term, *a*_*1*_ is the constant term, *a*_*2*_ represents the coefficient of the number incorporated, and *β* is the parameter to be estimated for the other control variables.

There may be selective bias and endogenous issues between digital engagement and mental health in older adults. The digital engagement of older people is a choice made on their circumstances, not randomly, so there is a problem of selective bias. Moreover, the individual characteristics of older adults can have associations with both their digital engagement and mental health, in which case ordinary least squares estimation introduces selective bias problems. To control the possible selectivity bias, this chapter uses propensity score matching (PSM) to measure the net effect of digital engagement on the mental health of older adults. The model divides the sample into an experimental group and a control group and matches the model with a propensity score for phased analysis to reduce the influence and interference of other factors. The specific model is as follows:

$${MHealth}_{i}={MHealth}_{0i}+({MHealth}_{1i}-{MHealth}_{0i})D_{i}$$ (2)

$$ATT=E({MHealth}_{1i}-{MHealth}_{0i}|D_{i}=1)$$ (3)

In Formula (2), Di is the treatment variable. When Di equals 1, it indicates that individual i belongs to the experimental group and participates in the intervention project. Conversely, when Di equals 0, it indicates that individual i belongs to the control group and does not participate in the intervention project. In this study, the core independent variables are divided into two groups. The experimental group comprises older adults with digital engagement, while the control group consists of older individuals without digital engagement. Eq (3) denotes the average treatment effect observed in the experimental group. It quantifies the net effect of digital engagement on the mental health of older adults.

Although the propensity score matching (PSM) method partially mitigates the selectivity bias among samples, it is limited to matching based on observable variables. Consequently, the issue of "invisible bias" arising from unobservable variables and potential endogenous problems, such as reverse causation, persists objectively. Theoretically, digital engagement among seniors can provide them with more self-information, facilitate communication with the external world, enhance emotional experiences, and improve their mental health. However, it is important to acknowledge that seniors with better mental health may exhibit greater digital engagement, while those with poorer mental health may struggle due to limited cognitive abilities. This reciprocal causal relationship between digital engagement and the mental health of seniors significantly contributes to endogeneity problems. Additionally, certain objective yet difficult-to-measure variables may also influence the digital engagement and mental health of older adults. Instrumental variable methods, such as the two-stage least squares method (2SLS), can generally address such issues. In this study, the 2SLS method is employed to resolve the endogeneity problem in the model. The selection of instrumental variables should satisfy both correlation and exogenous criteria, meaning that the instrumental variable must be associated with the endogenous variable while also meeting the exogeneity condition. In this context, the provincial IPv4 ratio demonstrates a certain correlation with the digital engagement of older individuals but is unrelated to their mental health, making it an appropriate instrumental variable. This choice is based on two considerations. First, the regional IPv4 ratio serves as an indicator of the overall digital engagement level within the jurisdiction, which is closely related to the digital engagement of older individuals. Hence, it satisfies the correlation requirements of instrumental variables. Second, the regional IPv4 ratio does not directly effect the mental health of older adults, thus meeting the exogeneity conditions required for instrumental variables.

Digital engagement offers individuals additional channels to expand their social networks. Specifically, for older adults, digital engagement has the potential to not only provide improved emotional support but also enhance social relationships among this demographic. Can digital engagement play a role in mitigating negative emotions experienced by the older adults in the digital realm and contribute to their emotional well-being? Furthermore, can it facilitate connections between older individuals and their friends and family, granting them access to more effective social support? To investigate the association of digital engagement on the mental health of older individuals, this study constructs a mediating effect model to explore potential mechanisms.

The construction of the mediating effect model involves three steps. First, Eq (4) demonstrates the regression of the explanatory variables on the core explanatory variables, where its represents the digital engagement level of the older adults and its impact on their mental health status. Second, Eq (5) showcases the regression of the intermediary variable (M) on the core explanatory variable. Finally, Eq (6) depicts the simultaneous regression of the explanatory variable and the mediator variable on the core explanatory variable. The complete mediation effect model in this paper comprises the following equations:

$$MHealth=\lambda +cDigital+\varepsilon_{1}$$ (4)

$$M=\lambda +aDigital+\varepsilon_{2}$$ (5)

$$MHealth=\lambda +c\prime \;Digital+bM+\varepsilon_{3}$$ (6)

## 3. Results

### 3.1 Benchmark regression results

Table 2 presents the findings of a stepwise regression analysis investigating the relationship of digital engagement on depression among older adults. The analysis systematically incorporates a set of control variables to explore the relationship. Successive models (model 1 to 5) progressively include variables representing individual characteristics, individual experiences, family characteristics, and community characteristics of older individuals. Across all models, the coefficients for numerical integration consistently exhibit positive values, indicating a statistically significant improvement in the mood of older adults at a 1% significance level. These results from the benchmark regressions provide compelling evidence supporting the significant positive relationship between digital engagement and the emotional well-being of older adults.

**Table 2:** Relationship between digital engagement and depression of older adults (OLS).

	Depression				
	(1)	(2)	(3)	(4)	(5)
Digital engagement	-2.178***	-1.827***	-1.694***	-1.706***	-1.620***
	(0.101)	(0.116)	(0.116)	(0.115)	(0.115)
Age		0.020***	0.014***	0.014***	0.014***
		(0.005)	(0.005)	(0.005)	(0.005)
Gender		0.026	0.008	-0.003	0.014
		(0.066)	(0.065)	(0.065)	(0.065)
Marriage		-0.514***	-0.481***	-0.476***	-0.494***
		(0.076)	(0.076)	(0.076)	(0.075)
Registration		0.104	0.045	0.042	0.011
		(0.071)	(0.070)	(0.070)	(0.069)
Major accidents			0.842***	0.833***	0.812***
			(0.086)	(0.086)	(0.085)
Childhood hunger			0.727***	0.720***	0.755***
			(0.069)	(0.069)	(0.069)
Parents survive			-0.423***	-0.412***	-0.386***
			(0.098)	(0.098)	(0.098)
Child support				-0.133	-0.131
				(0.095)	(0.095)
Home light				0.397***	0.371***
				(0.103)	(0.102)
Community policing					-0.430***
					(0.040)
Constant	9.511***	8.240***	8.391***	8.448***	10.006***
	(0.051)	(0.397)	(0.405)	(0.412)	(0.437)
R-squared	0.049	0.057	0.082	0.084	0.096
N	9,278	9,278	9,278	9,278	9,278

Note

*** p<0.01

** p<0.05

* p<0.1. Robust standard errors are in parentheses.

The control variables in Table 2 indicate a significant association between increasing age and worsening depression among older adults, with a significance level of 1%. The results concerning marital status reveal that married older individuals generally exhibit better emotional well-being than their unmarried counterparts. Based on the experiences of older individuals, it is evident that a major accident within the past year significantly and adversely affects their emotional state, often resulting in heightened negative emotions. Furthermore, early life experiences play a role in the emotional conditions of older individuals, as older adults who experienced childhood hunger tend to exhibit poorer emotional well-being than those who did not experience such circumstances. Additionally, older adults who had surviving parents at the age of 10 tend to have better emotional conditions in their later years. At the household level, receiving child support is associated with better mood among older individuals, as indicated by a negative significant relationship at a 1% level of significance. Moreover, older adults residing in well-lit home environments tend to experience better emotional well-being. Finally, at the community level, the emotional state of the older adults is positively influenced by a safer security situation within the community.

Table 3 presents the benchmark regression results illustrating the relationship between digital engagement and cognitive scores of older adults. Models (6) to (10) progressively include variables pertaining about individual characteristics, early life experiences, family characteristics, and community characteristics of older individuals. Notably, the coefficient values associated with digital engagement exhibit a statistically significant positive effect at a significance level of 1%, indicating a substantial enhancement in the cognitive ability of the older adults through digital engagement.

**Table 3:** Relationship between digital engagement and cognitive ability of older adults (OLS).

Variables	Cognitive ability				
	(6)	(7)	(8)	(9)	(10)
Digital engagement	2.803***	1.506***	1.501***	1.510***	1.497***
	(0.085)	(0.097)	(0.098)	(0.098)	(0.098)
Age		-0.100***	-0.097***	-0.098***	-0.098***
		(0.005)	(0.005)	(0.005)	(0.005)
Gender		0.238***	0.240***	0.258***	0.255***
		(0.060)	(0.060)	(0.060)	(0.060)
Marriage		0.301***	0.282***	0.272***	0.274***
		(0.074)	(0.073)	(0.073)	(0.073)
Registration		-0.757***	-0.749***	-0.750***	-0.746***
		(0.065)	(0.064)	(0.065)	(0.064)
Major accidents			-0.694***	-0.679***	-0.675***
			(0.086)	(0.086)	(0.086)
Childhood hunger			-0.118*	-0.108*	-0.113*
			(0.061)	(0.061)	(0.061)
Parents survive			0.281***	0.273***	0.269***
			(0.096)	(0.096)	(0.096)
Child support				0.309***	0.308***
				(0.094)	(0.094)
Home light				-0.347***	-0.343***
				(0.093)	(0.093)
Community policing					0.066*
					(0.038)
Constant	11.274***	18.979***	18.745***	18.550***	18.312***
	(0.055)	(0.398)	(0.403)	(0.407)	(0.429)
R-squared	0.087	0.151	0.160	0.162	0.163
N	9278	9278	9278	9278	9278

Note

*** p<0.01

** p<0.05

* p<0.1. Robust standard errors are in parentheses.

By considering the outcomes presented in both Tables 2 and 3, we can conclude that digital engagement not only significantly enhances the emotional well-being of older adults but also effectively improves their cognitive capabilities. Consequently, it can be inferred that digital engagement has a noteworthy positive effect on the mental health of older individuals.

The results of the control variables presented in Table 3 reveal several noteworthy findings. The coefficient values for age indicate a significant negative effect, suggesting a decline in cognitive performance among the older adults as they advance in age. Furthermore, it appears that older men exhibit superior cognitive ability when compared to older women.

From a physiological standpoint, an intriguing "health-survival" paradox emerges between men and women at old age [26]. Despite women having a higher average life expectancy, men tend to enjoy better overall health. Additionally, older women often face a higher burden of familial care responsibilities and experience greater vulnerability in society compared to their male counterparts [27]. Moreover, female older individuals tend to possess heightened emotional sensitivity, requiring more time to adapt and regulate negative emotions in response to environmental changes, ailments, and other risks. Consequently, their mental health tends to be comparatively poorer than that of men.

In contrast, the coefficient value of marriage demonstrates a significant positive association, indicating that married older individuals tend to exhibit better cognitive abilities. This finding may be attributed to the intricate social transformations occurring in China. With the diminishing functional and maintenance responsibilities of families, mutual support and assistance between older couples appear to have a beneficial effect on their health [28].

In conclusion, the analysis of the control variables provides valuable insights into the cognitive performance of older individuals. The results highlight the influence of age, gender, marital status, and social factors on cognitive abilities, emphasizing the need for a comprehensive understanding of these intricate relationships in gerontology research.

The cognitive ability of rural older individuals is inferior to that of their urban counterparts. This discrepancy can be attributed to several factors. First, urban older individuals enjoy better social welfare and overall economic strength than their rural counterparts, resulting in improved material security in their daily lives. Second, urban areas provide better accessibility and quality of medical services, enabling urban older individuals to receive superior healthcare and effectively address health problems and disease prevention risks [29]. Third, urban older individuals have more opportunities for communication and engagement with the outside world through participation in community activities, whereas rural areas often lack such collective activities. This is especially true for a significant number of left-behind older individuals who experience limited social interactions and poorer health conditions. Furthermore, negative experiences such as major accidents and childhood hunger can detrimentally effect cognitive performance at old age. Conversely, having both parents alive at the age of ten positively influences cognitive ability in older individuals. In terms of family characteristics, child support has a significant positive effect on the cognitive ability of older individuals, demonstrated at a level of significance of 1%. On the other hand, living in dimly lit homes significantly hampers the maintenance of cognitive ability on older individuals. Last, community policing initiatives have a positive relationship on the cognitive ability of older individuals.

### 3.2 Robustness test

To ensure the robustness of the benchmark regression results, we employed the substitution variable method for regression analysis. Table 4 presents the incorporation of numbers into the core explanatory variable, along with their respective layering techniques, followed by their substitution into the model. Specifically, Models (11), (12), and (13) investigate the effects of digital devices, digital use, and digital literacy on depression levels in older adults, respectively.

**Table 4:** Robustness test (OLS).

Variables	Depression		
	(11)	(12)	(13)
Digital devices	-1.176***		
	(0.087)		
Digital use		-1.329***	
		(0.090)	
Digital literacy			-0.229***
			(0.074)
Other variables	Yes	Yes	Yes
Constant	9.243***	9.525***	8.166***
	(0.420)	(0.425)	(0.428)
R-squared	0.094	0.099	0.077
N	9278	9278	9278

Note

*** p<0.01

** p<0.05

* p<0.1.

Robust standard errors are in parentheses.

The regression results from the four models consistently demonstrate that, despite using different dimensions to measure digital engagement and stratifying the digital engagement variables, number integration yields a significantly positive effect at a 1% significance level. This finding further supports the notion that digital engagement has a substantial positive effect on depression among older individuals.

Moreover, community policing has been found to have a positive effect on the cognitive ability of older adults.

Table 5 presents the robustness analysis of the association of digital engagement and cognitive ability of older individuals. The study stratifies the core explanatory variable of digital engagement and incorporates it into the respective models. Model (14), Model (15), and Model (16) assess the effects of digital devices, digital use, and digital literacy on the cognitive ability of older adults, respectively. The regression results from these four models consistently demonstrate a significant positive association between digital engagement and cognitive ability among older individuals, with statistical significance at the 1% level. These findings further confirm the substantial positive relationship between digital engagement and the cognitive ability of older individuals, even when employing different dimensions to measure digital engagement and stratifying the digital engagement variables.

**Table 5:** Robustness test (OLS).

Variables	Cognitive ability		
	(14)	(15)	(16)
Digital devices	0.572***		
	(0.073)		
Digital use		0.545***	
		(0.067)	
Digital literacy			1.080***
			(0.077)
Other variables	Yes	Yes	Yes
Constant	19.644***	19.638***	18.657***
	(0.421)	(0.422)	(0.410)
R-squared	0.149	0.149	0.167
N	9278	9278	9278

Note

*** p<0.01

** p<0.05

* p<0.1.

Robust standard errors are in parentheses.

### 3.3 Endogenous treatment

The preliminary analysis suggests that digital engagement significantly contributes to the mental well-being of older individuals. However, it is important to consider that various factors can influence both the mental health of older people and their level of digital engagement. Furthermore, more digital engagement is associated with better mental health among older individuals. Consequently, relying solely on ordinary least squares (OLS) regression may yield inaccurate correlation coefficients in models (5) and (10). To address this issue, instrumental variables are employed for two-stage least squares (2SLS) estimation. In this study, the IPv4 ratio at the regional (province) level is employed as the instrumental variable.

Table 6 presents the regression results of the IPv4 scale as an instrumental variable using 2SLS approach. The first column of Table 6 reveals that: the first stage F value is 411.54, indicating a strong relationship between the instrumental variable and the endogenous variable; the instrumental variable t value is 17.85, further confirming its robust explanatory power; and the Wald F statistic is 821.80, surpassing the critical value of 16.38 suggested by Stock and Yogo (2002) [30], thereby indicating the absence of a weak instrumental variable problem.

**Table 6:** Results of the endogenous treatment (2SLS).

Variables	Depression	Cognitive ability
Digital engagement	-5.740***	1.019**
	(0.666)	(0.411)
Other variables	Yes	Yes
Constant	15.589***	18.960***
	(0.986)	(0.683)
The first stage of the F value test	411.54***	411.54***
The value of the tool variable T	17.85***	17.85***
Wald F statistic	759.47***	1350.01***
N	9278	9278

Note

*** p<0.01

** p<0.05

* p<0.1.

Robust standard errors are in parentheses.

The estimates obtained from the instrumental variables approach demonstrate a significant positive effect of digital engagement on the emotional well-being of older adults. Turning to the second column of Table 6, we observe that the first stage F value test and the instrumental variable t value also indicate the strong explanatory power of the instrumental variable. Additionally, the Wald F statistic is 1350.01, providing further evidence of the absence of instrumental variable issues. These results imply that digital engagement has a substantial positive effect on the cognitive ability of older adults. Specifically, each unit increase in digital engagement among the older is associated with a 1.019-point increase in cognitive ability scores.

In summary, the findings reveal that digital engagement significantly influences the emotional well-being and cognitive ability of older adults. Furthermore, digital engagement has a noteworthy effect on reducing depression among older individuals.

### 3.4 Counterfactual test based on propensity score matching

This section utilizes propensity score matching (PSM) to address issues related to self-selection bias and to estimate the net effect of digital engagement on older people’s mental health. Specifically, the chapter focuses on the digital engagement of older individuals. The control group comprises older individuals who lack access to digital devices and do not engage in digital activities. Meanwhile, the treatment group consists of older individuals who have achieved varying degrees of digital engagement. Three commonly employed PSM methods are utilized: nearest neighbor matching, radius matching, and kernel matching. The sample balance test based on nearest neighbor matching shows that compared with the results before matching, the standard deviation (% bias) of all variables after matching is less than 10%, and the difference in sample features is eliminated by a large margin, indicating that the matching results can better balance the data.

Table 7 presents the estimates of propensity score matching for depression levels and cognitive ability in older adults. In the depression matching equation, the ATT value of digital engagement based on the nearest neighbor matching estimation is -0.7577, which is significant at the 1% level, indicating that digital engagement can significantly reduce the depression symptoms of older adults. The study continues to use radius matching and kernel matching for estimation, and the results are consistent with those of nearest neighbor matching. In the cognitive ability matching equation, the ATT value of digital engagement based on the nearest neighbor matching estimation is 0.2178, which is significant at the 1% statistical level, indicating that digital engagement can significantly improve the cognitive ability of older adults. The study continues to use radius matching and kernel matching for estimation, and the results are consistent with those of nearest neighbor matching. Therefore, digital engagement is consistent in the depression and cognitive ability equation, and the direction of the coefficient value is consistent with the estimation result of the benchmark regression. This conclusion further verifies that digital engagement can significantly improve the mental health of older people.

**Table 7:** Retest of the propensity score matching method based on counterfactual inference.

Variable name	Matching method	ATT	Standard deviation	T statistics
Depression	Nearest Neighbor Matching (1:4)	-0.7577	0.0864	-8.77***
	Radius Matching (0.003)	-0.7651	0.0956	-8.01***
	Kernel Matching (Default)	-0.7989	0.0745	-10.72***
Cognitive ability	Nearest Neighbor Matching (1:4)	0.2178	0.0831	2.62***
	Radius Matching (0.003)	0.2056	0.0892	2.3***
	Kernel Matching (Default)	0.3118	0.0721	4.32***

Note

*** p<0.01

** p<0.05

* p<0.1.

Based on the analysis of the benchmark regression and endogenous treatment mentioned above, the study progressively incorporated control variables and employed the 2SLS method to address endogeneity, along with the propensity score matching method to mitigate self-selection bias. The findings consistently demonstrate that digital engagement exhibits robustness in significantly enhancing depression among the older adults and consistently promoting their cognitive abilities. Therefore, it can be cautiously concluded that digital engagement plays a significant role in enhancing the mental health of older adults.

### 3.5 Heterogeneity analysis

The results indicate that digital engagement plays a crucial role in enhancing the mental well-being of older adults. However, it is important to note that the relationship of digital engagement varies across demographic groups. Table 8 presents the outcomes of heterogeneity tests conducted to assess the association of digital engagement on the level of depression among older adults belonging to various groups with chronic diseases. Notably, the positive effect of digital engagement on alleviating depression and enhancing cognitive ability is more pronounced among older adults without chronic diseases.

**Table 8:** Effect of digital engagement on the mental health of older adults with chronic diseases.

variables	Depression		Cognitive ability	
	chronic disease	Nonchronic disease	chronic disease	Nonchronic disease
Digital engagement	-1.591***	-1.994***	1.305***	2.024***
	(0.134)	(0.223)	(0.113)	(0.202)
Other variables	Yes	Yes	Yes	Yes
Constant	10.735***	9.252***	18.932***	16.949***
	(0.507)	(0.855)	(0.500)	(0.838)
R-squared	0.098	0.091	0.164	0.169
N	6879	2399	6879	2399

Note

*** p<0.01

** p<0.05

* p<0.1. Robust standard errors are in parentheses.

Table 9 examines the association of digital engagement with the mental well-being of older individuals across various age groups. The older participants were categorized into two groups based on age differences: young older individuals aged 60–69 years and middle-aged to older individuals aged 70 years and above. Regression analysis results revealed that within the sub-samples of these two age groups, digital engagement had a more pronounced effect on depression among young older individuals. Additionally, it demonstrated a stronger effect on cognitive performance in older individuals.

**Table 9:** Effect of numerical integration on the mental health of older adults of different ages.

variables	Depression		Cognitive ability	
	Higher age	Lower age	Higher age	Lower age
Digital engagement	-0.938***	-2.009***	1.776***	1.518***
	(0.185)	(0.149)	(0.196)	(0.109)
Other variables	Yes	Yes	Yes	Yes
Constant	9.507***	6.575***	21.183***	14.562***
	(0.740)	(1.268)	(0.873)	(0.920)
R-squared	0.073	0.108	0.138	0.100
N	4251	5027	4251	5027

Note

*** p<0.01

** p<0.05

* p<0.1.

Robust standard errors are in parentheses.

### 3.6 Further analysis

We assessed loneliness among older adults by examining their self-reported feelings of loneliness over the past week, and we evaluated the support provided by their relatives and friends through measures of relative support and friend support. Through the construction of a mediating effect model, we investigated the relationship between digital engagement and the mental health of older adults.

Table 10 presents the test results of loneliness as a mediating variable. The first column indicates the association of digital engagement and the loneliness experienced by older individuals. The coefficient value for digital engagement is negative and statistically significant at the 1% level, indicating that digital engagement significantly reduces loneliness of older people.

**Table 10:** Mechanistic tests at the level of loneliness of older adults.

variables	loneliness	Depression	Cognitive ability
digital engagement	-0.159***	-1.301***	1.500***
	(0.018)	(0.109)	(0.107)
loneliness		2.009***	0.023
		(0.062)	(0.060)
Other variables	Yes	Yes	Yes
Constant	4.706***	8.587***	18.296***
	(0.068)	(0.407)	(0.399)
R-squared	0.064	0.189	0.163
N	9278	9278	9278

Note

*** p<0.01

** p<0.05

* p<0.1.

Robust standard errors are in parentheses.

In the second column, both loneliness and digital engagement were included in the regression model to examine their effects on the mood of elderly individuals. The results reveal that the coefficient value for digital engagement continues to significantly reduce the level of depression among older individuals at the 1% level of statistical significance. Additionally, loneliness has a significant positive effect on the depression level of older individuals at the 1% level of statistical significance.

The third column investigates the effects of digital engagement and loneliness on the cognitive ability of older adults. The findings indicate that loneliness does not significantly effect the cognitive ability of older adults. These findings confirm the validity of the proposed mechanism in this study, which suggests that digital engagement can indirectly affect the depression level of older individuals by reducing their feelings of loneliness.

Table 11 presents the test results pertaining to family and friend support as an intermediary variable. In the first column, we observe the association of digital engagement and the support received by the older adults from their relatives and friends. The coefficient value of digital engagement is positive and statistically significant at the 1% level, indicating that digital engagement significantly promotes older individuals to obtain greater support from their relatives and friends.

**Table 11:** Mechanistic tests at the level of social support of older adults.

Variables	Social support	Depression	Cognitive ability
Digital engagement	1.051***	-1.406***	1.437***
	(0.076)	(0.115)	(0.107)
Social support		-0.204***	0.057***
		(0.016)	(0.015)
Other variables	Yes	Yes	Yes
Constant	2.850***	10.587***	18.151***
	(0.282)	(0.426)	(0.398)
R-squared	0.048	0.112	0.164
N	9278	9278	9278

Note

*** p<0.01

** p<0.05

* p<0.1.

Robust standard errors are in parentheses.

Moving on to the second column, we incorporate family and friend support as well as digital engagement into the regression model and assess their effect on the mental health of older individuals. The results demonstrate that the coefficient value of digital engagement continues to significantly decrease the level of depression among older individuals, also at the 1% statistical level. Furthermore, the support provided by relatives and friends exhibits a significant negative effect on the depression level of older individuals, again at the 1% statistical level.

In the third column, we examine the effects of digital engagement and family and friend support on cognitive performance of older adults. The results also reveal statistically significant effects at the 1% level. These findings establish the proposed mechanism of family and friend support, whereby digital engagement indirectly affects the depression level and cognitive ability of older individuals by enhancing the support they receive from their relatives and friends.

## 4. Discussions

The positive effect of digital engagement on the mental health of the older adults is evident through its ability to alleviate depression and enhance cognitive abilities. According to socio-emotional selectivity theory, in contrast to younger individuals, older adults are often acutely aware of the finite nature of their remaining time, leading them to prioritize immediate emotional gratification over long-term goals. While both time orientations coexist among older adults, advancing age tends to strengthen the inclination towards a present-focused mindset. Digital engagement of older adults transcends mere device ownership, encompassing the acquisition of essential digital skills. Embracing digital engagement thus equips older adults to access knowledge and novel experiences while also attending to their emotional well-being and pursuing satisfaction.

Digital engagement not only facilitates emotional fulfillment for older adults but also helps maintain their cognitive functioning, thus reducing the likelihood of dementia to some extent. Hence, this article establishes that digital engagement can yield mental health benefits for older individuals. Consequently, supporting and enhancing digital engagement among the older adults is of utmost importance in safeguarding their mental well-being.

For older individuals, the level of mental health varies among different groups, leading to differences in the factors that affect their mental well-being. This indicates the presence of group heterogeneity. First, the positive effect of digital engagement on mental health is more pronounced among older individuals without chronic diseases. Those with chronic illnesses often experience distress due to their health condition, directing their attention toward their disease status and increasing their susceptibility to anxiety. Consequently, they may exhibit social avoidance behavior, and their emotional state tends to be poorer. As a result, the alleviating effect of digital engagement on depression is relatively weaker for older individuals with chronic diseases.

Second, digital engagement can effectively reduce depression in both young and older individuals, with a more substantial effect on the cognitive ability of middle-aged and older individuals. Younger seniors find it less challenging to embrace digital engagement, as they are less likely to abandon it due to technological barriers or learning disabilities. Hence, they can derive a more positive emotional experience from digital engagement, which contributes to alleviating depression. Conversely, the cognitive capacities of older individuals tend to decline with age. Consequently, digital engagement may prove more beneficial for improving their marginal cognitive abilities.

Mechanism analysis demonstrates that digital engagement has the potential to enhance mental health among older individuals by addressing feelings of loneliness and improving the support they receive from relatives and friends. By facilitating social interaction and providing virtual network support, digital engagement effectively reduces loneliness and positively impacts emotional well-being. Digital engagement can play a positive role in the psychological well-being of older adults by reducing feelings of loneliness through enhanced social interaction and virtual network support. Firstly, digital engagement provides older adults with opportunities for social interaction and connection with others. Utilizing digital tools such as social media, video calls, and online communities, older adults can stay connected with family, friends, and others to share life experiences and emotions. This social interaction and connection not only help alleviate feelings of loneliness but also increase social support and improve psychological well-being. Secondly, digital engagement provides older adults with avenues to access various resources and information. Older adults can acquire knowledge, culture, and entertainment content through internet searches, online learning platforms, and e-books. Accessing these resources not only provides mental support and enriches the lives of older adults but also reduces feelings of loneliness and boredom, thereby promoting psychological well-being. Additionally, digital engagement enables older adults to join virtual communities and support networks. These communities and networks provide platforms for older adults to interact with like-minded individuals and those with similar interests. Through online social platforms and interest groups, older adults can make new friends, participate in discussions, share experiences, and receive support. Participation in these communities and networks helps to increase older adults’ social support networks, alleviate feelings of loneliness, and enhance levels of psychological well-being.

According to social support theory, individuals experience emotional support, practical assistance, and informational guidance through interactions and connections with others, leading to improved mental health outcomes [31]. Through digital engagement, older individuals gain access to platforms for communication and interaction, expanding their social networks and support systems. This integration offers additional avenues for seniors to engage in social interaction, enabling them to connect with family, friends, and others, share experiences, receive emotional support, and access practical assistance and information. Digital engagement offers older adults more channels to engage in social interactions, leading to increased social connections and a sense of belonging. Through digital technology, older adults can establish more stable and robust social relationships, thereby enhancing social support and contributing to the maintenance and improvement of mental well-being.

## 5. Conclusions

With the rise of an aging population and the rapid advancement of social digitization, this paper utilizes the 2018 CLASS data to examine the relationship between digital engagement and the mental health of older individuals, specifically focusing on depression and cognitive ability. The results reveal two key aspects. First, digital engagement proves to be effective in enhancing the mental well-being of older individuals. The conclusion remains valid even after conducting rigorous robustness tests and endogenous analyses. Second, the heterogeneity test indicates that the association of digital engagement and depression levels is more pronounced among younger older individuals without chronic diseases, whereas the effect on cognitive ability is more prominent among older age groups without chronic diseases. This suggests that when promoting digital engagement among older individuals, it is essential to consider equitable access across different age groups.

Moreover, the mechanism analysis demonstrates that digital engagement facilitates the improvement of older adults’ mental health by reducing feelings of loneliness and enhancing their social support networks.

The implications of this study are enlightening in assisting the digital engagement of the older adults and promoting their mental well-being. Recognizing that digital engagement benefits the mental health of older individuals, it is crucial to provide increased social support to aid their digital engagement and enhance their mental well-being. Simultaneously, efforts should be made to ensure that vulnerable groups within the older population are not further marginalized in the process of digital engagement, thereby mitigating mental health inequalities across different age groups. At the same time, it is necessary to acknowledge certain limitations in this study. In rural areas of China, many elderly individuals still face challenges related to the digital divide, lacking access to digital devices. This impediment makes it difficult for them to effectively enhance their mental well-being through digital integration. Therefore, seeking effective strategies to promote digital inclusion among rural elderly individuals and reduce the resulting health inequalities stemming from digital inequities constitutes both a limitation of this study and a crucial direction for future research.

## Supporting information

S1 Data (ZIP)
